# What are effective strategies for implementing trauma-informed care in youth inpatient psychiatric and residential treatment settings? A realist systematic review

**DOI:** 10.1186/s13033-017-0137-3

**Published:** 2017-05-11

**Authors:** Stephanie A. Bryson, Emma Gauvin, Ally Jamieson, Melanie Rathgeber, Lorelei Faulkner-Gibson, Sarah Bell, Jana Davidson, Jennifer Russel, Sharlynne Burke

**Affiliations:** 1 Portland State University, School of Social Work, 1800 SW 6th, Building ASRC 620G, Portland, OR 97207-0751 USA; 2 University of British Columbia, School of Social Work, 2080 West Mall, Vancouver, BC V6T 1Z2 Canada; 3 Mental Health, BC Children’s Hospital, 4500 Oak Street, Vancouver, BC V6H 3N1 Canada; 4 Children’s & Women’s Hospitals and Health Centre, 4500 Oak Street, Vancouver, BC V6H 3N1 Canada; 5 Child & Adolescent Mental Health & Concurrent Disorders Programs, BC Children’s Hospital, 4500 Oak Street, Vancouver, BC V6H 3N1 Canada

**Keywords:** Trauma-informed care, Trauma informed practice, Implementation science, Youth mental health, Inpatient psychiatric care, Residential care

## Abstract

**Background:**

Many young people who receive psychiatric care in inpatient or residential settings in North America have experienced various forms of emotional trauma. Moreover, these settings can exacerbate trauma sequelae. Common practices, such as seclusion and restraint, put young people at risk of retraumatization, development of comorbid psychopathology, injury, and even death. In response, psychiatric and residential facilities have embraced trauma-informed care (TIC), an organizational change strategy which aligns service delivery with treatment principles and discrete interventions designed to reduce rates of retraumatization through responsive and non-coercive staff-client interactions. After more than two decades, a number of TIC frameworks and approaches have shown favorable results. Largely unexamined, however, are the features that lead to successful implementation of TIC, especially in child and adolescent inpatient psychiatric and residential settings.

**Methods:**

Using methods proposed by Pawson et al. (J Health Serv Res Policy 10:21–34, 2005), we conducted a modified five-stage realist systematic review of peer-reviewed TIC literature. We rigorously searched ten electronic databases for peer reviewed publications appearing between 2000 and 2015 linking terms “trauma-informed” and “child*” or “youth,” plus “inpatient” or “residential” plus “psych*” or “mental.” After screening 693 unique abstracts, we selected 13 articles which described TIC interventions in youth psychiatric or residential settings. We designed a theoretically-based evaluative framework using the active implementation cycles of the National Implementation Research Network (NIRN) to discern which foci were associated with effective TIC implementation. Excluded were statewide mental health initiatives and TIC implementations in outpatient mental health, child welfare, and education settings. Interventions examined included: Attachment, Self-Regulation, and Competency Framework; Six Core Strategies; Collaborative Problem Solving; Sanctuary Model; Risking Connection; and the Fairy Tale Model.

**Results:**

Five factors were instrumental in implementing trauma informed care across a spectrum of initiatives: senior leadership commitment, sufficient staff support, amplifying the voices of patients and families, aligning policy and programming with trauma informed principles, and using data to help motivate change.

**Conclusions:**

Reduction or elimination of coercive measures may be achieved by explicitly targeting specific coercive measures or by implementing broader therapeutic models. Additional research is needed to evaluate the efficacy of both approaches.

**Electronic supplementary material:**

The online version of this article (doi:10.1186/s13033-017-0137-3) contains supplementary material, which is available to authorized users.

## Lifelong effects of childhood trauma

Traumatic experiences overwhelm a person’s psychological ability to cope and a person’s biological capacity to regulate involved stress hormones [1]. Trauma itself is thus a highly individualized construct which can vary from relatively discrete occurrences like natural disasters and auto accidents, to ongoing emotional abuse and neglect, to structural violence resulting from inequality, colonial practices, and war [2–4]. Of particular clinical concern are traumatic experiences that occur in childhood [5].

In a landmark longitudinal study in mental health epidemiology, Felitti and colleagues [6] examined how adverse childhood experiences (ACE) correlated with lifelong physical and mental health conditions. More than two-thirds of the study’s 17,000 participants reported experiencing at least one ACE, which included three types of childhood abuse (psychological, physical, and sexual abuse) and four categories of household dysfunction (exposure to caregiver substance abuse, mental illness, violent treatment of mother or stepmother, and criminal behavior within the household). Findings revealed a strong proportionate relationship between respondents’ ACE scores and subsequent lifelong medical and mental health pathology and early mortality rates [7].

Critical periods for brain development occur throughout childhood [8], making childhood trauma particularly consequential to developing brain structures involved in executive functions and adaptive stress responsivity [1, 9, 10]. Interdisciplinary studies have demonstrated that nurturing and supportive caregiver relationships provide a protective ‘buffer’ against the effects of childhood trauma through co-regulation of emotional stress response [11–13]. In other words, relational security can reduce the effects of childhood trauma that might otherwise result in maladaptive behaviors [14]. Nurturing relationships between children and caregivers mediate the successful development of neurobiological functions that involve decision-making, working memory, self- and social-awareness, and mood and impulse control [15–19].

## Trauma among children and youth in inpatient psychiatric and residential settings

Traumatic stress is now understood to be at the root of many common behavioral issues—both internalizing and externalizing—for which children and youth are psychiatrically hospitalized or placed in residential facilities [20–22]. Teicher and colleagues [23–25] identified neurobiological consequences of childhood traumatic stress, which include reduced volume in critical brain structures associated with learning, memory, and emotion regulation. Thus, children exposed to violence at home, for example, may exhibit short term symptoms of generalized anxiety, sleeplessness, nightmares, difficulty concentrating, high activity levels, increased aggression, and worry about safety. Long term effects may include major depression, suicide, substance abuse, physical health problems, problems in school, and behaviors which result in incarceration.

Moreover, evidence is emerging that the severity of traumatic exposure is correlated with clinical severity. Two recent studies utilizing the U.S. National Child Traumatic Stress Network’s (NCTSN) Core Data Set found significant dose–response relationships between type of trauma and behavior problems in a sample of clinic-referred youth (*n* = 11,028) aged 1 ½ to 18 years old [26] and between trauma exposure and level of impairment among youth in residential care (*n* = 525) compared to youth in nonresidential settings (*n* = 9942) [27].

Similarly, a recent chart review of 1433 consecutively psychiatrically hospitalized children and adolescents aged 3–18 [28] suggests the following: (1) sexual and physical abuse are common among hospitalized youth, with more than one-third of the sample indicating traumatic exposure; (2) a history of trauma increases cross-diagnostic comorbidity and length of stay; and (3) youth with substantiated sexual abuse were prescribed 30% more medication upon admission and more atypical antipsychotic medications over the course of admission than were their counterparts without sexual abuse histories—even those with physical abuse histories.

Authors of all abovementioned studies conclude, respectively, by recommending “a trauma-informed public health and social welfare approach to prevention, risk reduction, and early intervention for traumatized youth” [26]; “less restrictive…community-based trauma-informed interventions” [27]; and “trauma-informed treatment in psychiatric hospital settings” [28].

## The need for trauma informed care in youth settings

Caring and supportive social environments that promote adaptive and relational caregiver responses to the behavioral and neurobiological sequelae of trauma appear to provide co-regulation of stress responsivity for children with histories of adversity [3, 9, 29]. Co-regulation of stress responsivity thus fosters developmental safety [4, 14, 30], making trauma-informed approaches particularly important in residential and in-patient environments.

In contrast to *trauma specific treatments* which use direct counseling techniques and interventions to reduce trauma symptoms (e.g., Seeking Safety or Trauma-Focused Cognitive Behavioral Therapy), *trauma*-*informed care or practice* (TIC or TIP) is more ambitious, aiming to transform entire systems of care by embedding an understanding of traumatic stress response “in all aspects of service delivery and plac[ing] priority on the individual’s safety, choice, and control” [31]. This philosophy aims to create a treatment culture of nonviolence, learning, and collaboration in which a universal precautions approach is highlighted in all environmental and interpersonal interactions.

Such universal precautions are assumed with regard to potentially traumatizing practices such as seclusion and restraint. Seclusion refers to the involuntary confinement of a child in a room or isolated area from which they may not leave. Restraint is the use of physical, mechanical, or chemical means to prevent a child’s physical mobility. Despite their historical use to manage “harm to self or others,” these practices may themselves be harmful, with documented cases of injury and death [32–34]. Indicia from Cochrane Collaboration reviews are unequivocal about the continued use of seclusion and restraint for adults with mental illness [35]:

No controlled studies exist that evaluate the value of seclusion or restraint in those with serious mental illness. There are reports of serious adverse effects for these techniques in qualitative reviews. Alternative ways of dealing with unwanted or harmful behaviours need to be developed. Continuing use of seclusion or restraint must therefore be questioned from within well-designed and reported randomised trials that are generalisable to routine practice.

Importantly, Lebel et al. [33] report that restraint and seclusion continue to be used on children, adolescents, and youth in residential settings at higher rates than on adults in care, “often with deleterious effects” (170). Initiatives aimed at reducing seclusion and restraint can be practiced within an overall TIC framework; however, TIC extends far beyond the reduction of seclusion and restraint use and into the overarching culture of safety within an organization.

## Implementing trauma-informed care: need for systematic review

Curiously, although a growing body of research documents detrimental lifelong impacts of childhood traumatic stress—and a growing chorus of voices demands trauma-informed approaches in community, inpatient, and residential treatment centers—the science regarding the *implementation* of trauma-informed care among youth in out of home settings is modest. In their recent review of seclusion and restraint reduction interventions with pretest and posttest designs, Valenkamp et al. [36] characterized this body of research in their title as “an undeveloped area,” locating only two models (Collaborative Problem Solving and Comprehensive Behavioral Management) with sufficient empirical evidence to merit inclusion in their review. Authors underscored the absence of randomized controlled trials testing these interventions. Moreover, Chandler [37] articulated the need to examine “critical factors that facilitate successful adoption of trauma-informed treatment across units that vary in location, size, and patient populations” (p. 370).

Given the discrepancy between high rates of traumatic stress among children and adolescents in psychiatric and residential facilities, along with a dearth of experimental research demonstrating how to reduce violent and coercive practices in such settings, we elected to conduct a systematic review of literature. The overarching aim of this project was to examine, systematically, the available scholarly literature on trauma-informed care in psychiatric inpatient and residential programs for youth. Foremost in our minds was the realist dilemma described by Pawson and colleagues [38]: “In health services…we are dealing with complex social interventions which act on complex social systems…These are not magic bullets which will always hit their target, but programmes whose effects are crucially dependent on context and implementation” (S1:21).

Within this implementation context, we posed the following realist review question of trauma-informed care: *What is it about trauma*-*informed care that works, for whom, in what circumstances, in what respects, and why?*

## Methods

Using methods proposed by Pawson and colleagues [38], we conducted a five-stage realist systematic review of peer reviewed literature on trauma-informed care in youth inpatient psychiatric and residential settings. The five stages included: (1) clarifying our scope; (2) searching for evidence; (3) appraising primary studies and extracting data; (4) synthesizing evidence and drawing conclusions; and (5) implementing and evaluating recommendations with stakeholders. As the U.S. Congress established the National Child Traumatic Stress Network in 2000, we delimited our search to the intervening years since its establishment, reasoning that 15 years would provide a sufficient period within which to test the effectiveness of TIC models and interventions. We did not publish a review protocol.

## Search terms and strategy

**Fig. 1:**
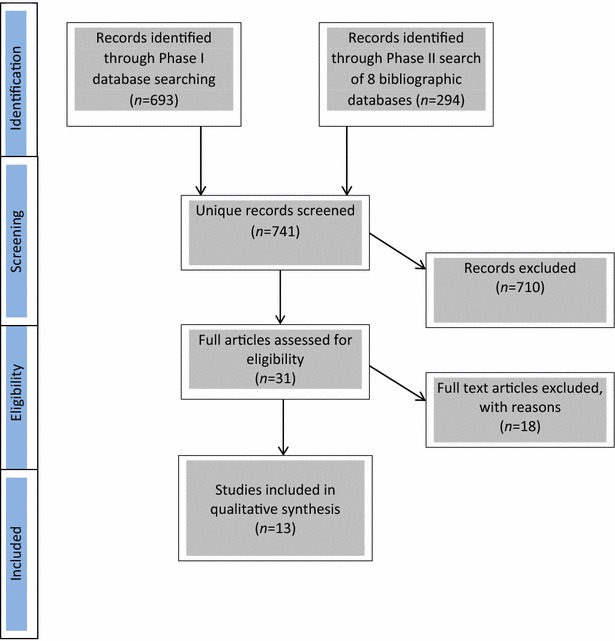
PRISMA flow diagram of study selection

Our search strategy was conducted in two phases (see Fig. 1).

In Phase 1, the second author (EG) conducted searches of all search engines and full text databases available through the University of British Columbia library, using terms “trauma-informed” and “child*” or “youth,” plus “inpatient” or “residential” plus “psych*” or “mental.” This search, conducted in April 2015, produced 693 results. All abstracts were reviewed for relevance using predetermined inclusion criteria (see next section), and an initial selection of articles was made.

In Phase 2, conducted in mid-May within a 5-day period, the first author (SB) performed searches on the following eight bibliographic databases using the EBSCO Host interface: (1) Academic Search Complete; (2) Cumulative Index to Nursing and Allied Health (CINAHL); (3) Education Source; (4) Educational Resources Information Centre (ERIC); (5) MEDLINE (Ovid); (6) Psych Articles; (7) PsycINFO; (8) Social Work Abstracts; and (9) Social Sciences Citation Index (SSCI). Searches produced 294 abstracts published within a defined ‘published within’ range of 1st January, 2000–15th May, 2015.

## Inclusion and exclusion criteria

All abstracts (n = 31) which described system-wide implementations of trauma-informed care were selected for in-depth review. Upon review, we excluded statewide or provincial mental health initiatives and TIC implementations exclusively in outpatient mental health, child welfare, and education settings, restricting results to those interventions and initiatives implemented in inpatient and residential settings only. Articles were included in the review if the initiative or intervention: (1) involved a change in organizational milieu; (2) was explicitly described as involving a “trauma-informed” approach; and (3) had been evaluated, even preliminarily, using predetermined measures. We read but excluded gray literature from the systematic review and ultimately selected thirteen (n = 13) peer-reviewed articles, whose bibliographies we scrutinized for additional citations. These articles are listed in Table 1. They include but are not limited to examination of the following trauma informed care models: the Attachment, Self-Regulation, and Competency Framework (ARC); the Six Core Strategies (6CS); Collaborative Problem Solving (CPS); the Sanctuary Model; Risking Connection (RC); and the Fairy Tale Model.

**Table 1:** Articles included in systematic review

Articles included	**Model**	**Setting and country**
Azeem et al. [40]	Six core strategies	Inpatient-U.S.
Brown et al. [41]	Risking connection	Congregate care-U.S.
Caldwell et al. [42]	Six core strategies	Residential-U.S.
Deveau and Leitch [43]	Restraint reduction meeting	Residential-UK
Goetz and Trujillo [44]	Patient-focused intervention	Residential & Inpatient-U.S.
Greene et al. [45]	Collaborative problem solving	Inpatient-U.S.
Greenwald et al. [20]	Fairy tale model	Residential-U.S.
Holstead et al. [47]	Quality plus program	Residential-U.S.
Hodgdon et al. [46]	Attachment, regulation, competency (ARC) model	Residential-U.S.
Hummer et al. [21]	Trauma-informed program self- assessment	Out of home incl residential- U.S.
Martin et al. [34]	Collaborative problem solving	Inpatient-U.S.
Rivard et al. [48]	Sanctuary model	Residential-U.S.
Russell et al. [49]	Devereaux’s safe & positive approaches	Residential-U.S.

## Data abstraction and framework analysis

The goal of this review was not to determine the most efficacious model of trauma informed care. In keeping with realist review methods, the goal was rather to consider, across a range of contexts, common elements of ‘successful TIC implementation’ among different patient groups. Accordingly, a range of methodologies were included. Studies varied in design from retrospective chart review, to pre- and post-test design, to prospective chart review. Specific trauma informed care initiatives included in this review fell into two main groups: (1) comprehensive, multi-component initiatives that were designed foremost to reduce use of seclusion and restraint (e.g., Six Core Strategies to Reduce Seclusion and Restraint), and (2) robust clinical TIC models focused on client symptomatology improvements and secondarily aimed at decreasing or eliminating use coercive practices in child and youth settings (e.g., Risking Connection, Fairy Tale Model; and Attachment, Self-Regulation, Competency model and others).

In keeping with realist review appraisal and extraction protocols, which differ from traditional systematic reviews in that they attend more to program theory than to research rigor [39], we designed a theoretically based evaluative framework. As this review concentrated specifically on the *implementation* of trauma-informed care, we used the active implementation cycles of the National Implementation Research Network (NIRN) as the foundation of our theoretical framework (see Fig. 2).

**Fig. 2:**
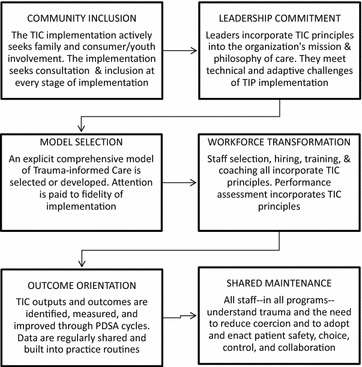
Initial program theory of trauma informed practice implementation

Given substantial overlap between the NIRN frameworks and the best known *implementation*-*informed* model for TIC in child and adult mental health settings, the National Association of State Mental Health Program Directors’ (NASMHPD) Six Core Strategies to Reduce Seclusion and Restraint (6CS), we then cross-matched the two frameworks (see Table 2).

**Table 2:**
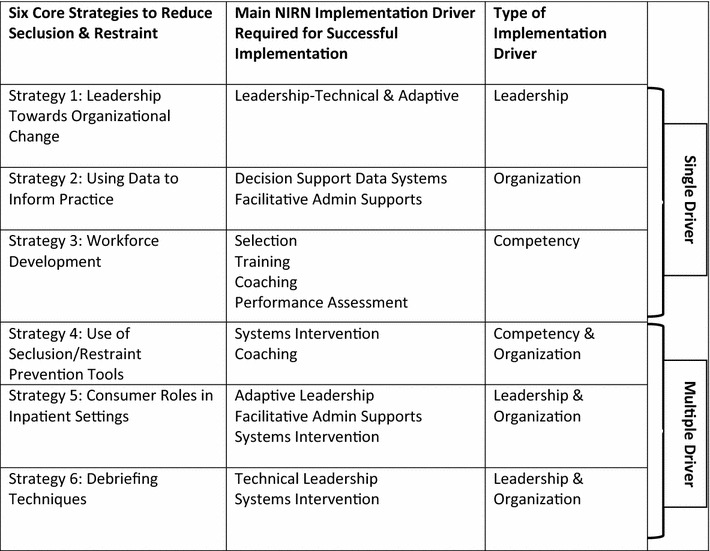
Crossmatch of NIRN implementation drivers with six core strategies

Next, we produced a hypothetical program theory of successful TIC based on the crosswalk of NIRN implementation drivers and the Six Core Strategies (Fig. 2), against which to test findings of the systematic review. Finally, we extracted evidence on the basis of relevance to our realist review research question, which differs from a typical PICO (Population, Intervention, Comparison, Outcome) question and instead asks, “What is it about trauma-informed care that works, for whom, in what circumstances, in what respects, and why?” Given the emphasis in realist review on the unique contributions of intervention *context*, *mechanism*, and *outcome*, we organized findings using the following categories: TIC Approach, Design, Context, Outcome and Implications (Table 3) and Mechanisms of Action (See Additional file 1: Table S1).

**Table 3:** TIC Model, design, context and outcomes in TIC implementation articles

Author (year), title	TIC approach	Design			Context	Outcome and implications
Azeem, Aujla, Rammerth, Binsfield, & Jones (2011) Effectiveness of Six Core Strategies based on Trauma Informed care in reducing seclusions and restraints at a child and adolescent psychiatric hospital	6 core strategies: (6CS-National Association of State Mental Health Program Directors-NASMHPD) Six core strategies are: Leadership Toward Org Change, Use of Data to Inform Practice, Youth & Family Inclusion, Workforce Development, S/R Prevention Tools, Debriefing	*Design* retrospective chart review of seclusion and restraint data for youths admitted (*n* = 458) July 2004-March 2007 (implementation of 6CS in final 6 mos) *Sample* examined S/R episodes for 458 youth (276 females/182 males) *Measures* age, race, gender, admission dx, LOS, admission status, seclusion, restraint episodes *Limitations* possible intervening variable: concurrent Dialectical Behavior staff training			26-bed adolescent unit (9-bed adol girl unit; 9-bed adol boy unit; 8-bed unit boys & girls, aged 6-12) *External factors:* Centers for Medicare and Medicaid Svcs, Joint Commission issued guidelines regarding use of seclusion and restraint	Marked reduction in use of seclusion and restraint—from 93 episodes (73 seclusions/20 restraints pre-6CS) to 31 episodes (6 seclusions/25 restraints) following implementation of Six Core Strategies Results achieved quickly and maintained over a period of time *Emphasized*: Leadership commitment Workforce transformation Outcome orientation
Brown, Baker, & Wilcox (2012) Risking Connection Trauma Training: A Pathway Toward Trauma-informed Care in Child Congregate Settings	Risking Connection (RC) trauma training: “The RC training teaches a trauma framework which asserts that childhood trauma…derail the trajectory of development in three critical areas—attachment, brain and nervous system, and self-capacities or self-regulation skills.”	*Design*: Study examined change in knowledge, beliefs, and self-reported behaviors pre- and post-Risking Connection (RC) training *Sample:* 261 child congregate care trainees over 17 months in 2008-2009 *Measures*: Risking Connection Curriculum Assessment, Trauma-informed Belief Measure, Staff Behavior in Milieu Measure *Limitations:* No observational data			Five youth congregate care agencies (residential, foster, etc.) serving children and youth with serious emotional and psychiatric problems *External factors*: NASMHPD’s & SAMHSA’s National Child Traumatic Stress Network (NCTSN) promotion of TIC in residential care	Three post-training measures indicated increase in (a) knowledge, (b) increase in beliefs favorable for TIC, and c) increase in self-report of TIC behavior Staff trained as trainers showed maintenance of positive changes in knowledge, beliefs, and behaviors *Emphasized*: Model selection Workforce transformation
Caldwell, et al. (2014) Successful Seclusion and Restraint Prevention Efforts in Child & Adolescent Programs	6 Core Strategies: (6CS-National Association of State Mental Health Program Directors-NASMHPD) Six Core Strategies are: Leadership Toward Org Change, Use of Data to Inform Practice, Youth & Family Inclusion, Workforce Development, S/R Prevention Tools, Debriefing	*Design*: Three site study of 6CS implementation. Qualitative description of 6CS implementation features + outcomes. *Sample*: Inpatient psychiatric facility with 52 youth beds; secure residential facility with 84 secure beds +48 therapeutic group home beds *Measures*: Mechanical & physical restraints, seclusions, focus groups with youth *Limitations*: Article profiles 3 different facilities’ implementation experiences. Descriptive; no methods reported			Site #1: Children’s Center with 52 youth psychiatric beds Site #2: Secure residential facility for youth with serious emotional disturbance Site #3: State of CT largest intensive residential program 100 male youth beds *External factors*: Part of larger national Building Bridges initiative, which sought to integrate the principles of trauma-informed care in residential and community settings	In Site #1: Between 2005 and 2013, mechanical restraints were 100% eliminated; restraint was reduced by 87%; seclusion reduced by 67% In Site #2: Restraints reduced from 49 in January 2012 to 1 in 2014 In Site #3: Restraint reduced by 75% between 2011 and 2013 *Emphasized*: Leadership commitment Workforce transformation Community inclusion
Deveau & Leich (2014) The impact of restraint reduction meetings on the use of restrictive physical interventions in English residential service for children and young people	Post Restraint Reduction Meetings (RRM): RRM are routine staff meetings to analyze/evaluate Restrictive Physical Intervention use; after initial training, they ideally occur within 72 h of each restraint episode	*Design*: Longitudinal pre-post intervention design examined impact of RRM on frequency of restraint *Sample*: 10 residential/Children’s Home settings *Measures*: Type, restrictiveness, length of time, & frequency of Restrictive Physical Interventions (RPI) *Limitations*: Intervention fidelity not monitored; confounding variables not assessed			UK children’s homes & residential full-time homes for looked after children and children with behavioral & emotional disturbance (BESD) *External factors*: Seclusion and restraint reduction measures in U.S.	Reduced mean frequency of Restrictive Physical Interventions pre- to post-intervention by 31.6% Greatest reduction in most restrictive supine floor restraints *Emphasized*: Workforce transformation
Goetz & Trujillo (2012) A change in culture: Violence prevention in an acute behavioral health setting	Patient-Focused Intervention (PFI) Model: Nine component model which includes TIC, aggression management, code event review, leadership involvement, quality feedback, recovery orientation, patient assessment, education, collaboration	*Design:* Pre-posttest, nonequivalent groups *Sample*: Adults & adolescents admitted during 5-year period from 2005–2010 *Measures*: S/R data, Code Gray episode data, staff injuries; staff safety survey *Limitations:* Gross reduction in Code Grey episodes reported but shown only in minutes of restraint usage			80 bed facility including two adol programs—19-bed adol female tx center; 15-bed acute psychiatric facility for youth 12–18 years old *External factors*: 2003 manual on reducing violence, & coercive measures by American Psychiatric Assn (APA), American Psychiatric Nurses Assn (APNA), National Assn of Psychiatric Health Systems (NAPHS), & American Hospital Assn (AHA)	Staff injuries decreased by 48% in first year of implementation Seclusion and restraint rates were reduced by 50%; 75% reduction in hours of S/R in first 2 years One full year after implementation, staff survey data showed improvement in 5 of 10 areas, including staff perception of aggression mgmt. program *Emphasized*: Leadership commitment Outcome orientation Shared maintenance
Greene, Ablon, & Martin (2006) Use of collaborative problem solving to reduce seclusion and restraint in child and adolescent inpatient units	Collaborative Problem Solving (CPS) Cognitive behavioral approach focused on adult-child decision making rather than teaching or motivating children to comply with adult directives	*Design:* Pretest-post- test; nonequivalent groups *Sample*: 100 children, mean age 9.14 years, were admitted during study period; 80% significant trauma histories; 95% admitted for severe out of control behavior *Measures*: Restraint episodes, staff and patient injuries *Limitations*: Could not control for intervening variables; generalizability may be affected by selecting unit with high pre-training # of S/R episodes			U.S. 13 bed, locked in-patient child psychiatry unit in Massachusetts children serving ages 3–14 years with average stay of 14 days *External factors*: Evidence of fatalities and other adverse outcomes following S/R use	Reduced S/R from 281 episodes recorded 9 mo pre-training to 1 incident recorded 15 mo post-training Reduced staff and patient injuries from an average of 10.8 per month to 3.3. per month *Emphasized*: Model selection Workforce transformation Outcome orientation Shared maintenance
Greenwald, Siradas, Schmitt, Reslan, Fierle, & Sande (2012) Implementing trauma-informed treatment for youth in a residential facility: First-year outcomes	Fairy Tale Model: (a) Designed for children, teens, & adults; (b) strong family and community component; (c) incorporates milieu treatment; (d) Included staff education and case mgmt.; (e) scripted interventions including each phase accompanied by telling of Fairy Tale; (f) model encouraged adaptation to agency’s existing culture	*Sample*: Youth ages 10-21 in facility between 2008-2009 (n = 53) *Measures*: PTSD sx, presenting problems, time to discharge, type of discharge H1: ↓ PTSD sx H2: ↓Primary presenting probs H3: ↓ Time in residential care H4: Rate of +discharges *Limitations*: Missing data on PTSD symptoms; delivery of individual therapy was uneven; no treatment fidelity measures; no comparison group due to AB design			Residential treatment facility serving children and youth aged 10–21 *External factors*: Western NY agency’s desire to address trauma component of clients’ problems. *Positive Peer Cult*ure, an evidence informed peer support model, was in place prior to implementation of trauma-informed treatment	Study found a 34% increase in problem reduction; 39% reduction in treatment time, double the rate of positive discharges *Emphasized*: Model selection Workforce transformation Outcome orientation
Hodgdon, Kinniburgh, Gabowitz, Blaustein, & Spinazzola (2013) Development and implementation of trauma-informed programming in youth residential treatment centers using the ARC framework	Attachment, Regulation, and Competency model: Framework for youth with complex trauma. Nine core building blocks: (1) Caregiver affect mgmt.; (2) attunement; (3) consistent response; (4) routines & rituals; (5) affect identification; (6) modulation; (7) affect expression; (8) executive functions; & (9) self-development Included elements of DBT	*Sample*: Young women aged 12–22 in two residential settings (*n* = 126) *Measures*: CBCL; UCLA PTSD Reaction Index; physical restraints *Limitations*: Statistically significant reductions in PTSD symptoms but modest clinical improvement, possibly due to uneven delivery of ARC model across programs			Two Massachusetts residential programs for young women ages 12–22, including an Intensive Residential Treatment Program and a residential school *External factors* Implementation of ARC model for this study was based on Fixsen et al. (2005) implementation stages. Funded by SAMSHA as part of National Child Traumatic Stress Initiative	Significant decrease in overall PTSD symptoms, and decrease in aggression, anxiety, attention problems, rule breaking, depression, thought problems, and somatic complaints based on CBCL scores There was a 50% reduction of use of restraint in the first 6 months and the trend continued downward ARC did not create any statistical difference in rates of PTSD numbing and avoidance *Emphasized* Leadership commitment Workforce transformation Outcome orientation
Holstead, Lamond, Dalton, Horne, & Crick (2010) Restraint reduction in children’s residential treatment facilities: Implementation at Damar Services	Resource Management Team focused on reducing restraint use. Training in verbal de-escalation. Each staff member experienced a restraint as part of training, and staff heard from patients who had experienced restraint. In 2008, agency declared itself restraint free	*Sample*: 215 youth with behavioral and developmental problems *Measures*: # of restraint, length of restraint, staff injury, client injury *Limitations*: Insufficient methodological information			Private non-profit residential setting for adults and children (*N* = 215) in Indianapolis, IN. Serves children with behavioral and developmental problems and many failed placements. Children and youth served have had as many as 30 failed prior placements	Reduced restraints from 5000 in 2004 (56 per child) to 786 in 2008 (3.66 restraints per child). Minutes in restraint decreased from 21 min avg to 12 min avg Staff injury rate decreased from .0199 to .0159 per person between 2004 and 2008. Client injury rate decreased from 307 to 145, or 3.49 injuries from restraint to .68 injuries per person *Emphasized* Leadership commitment Workforce transformation
Hummer, Dollard, Robst, & Armstrong (2010)	*Creating Trauma*-*informed Care Environments Curriculum*	*Sample*: Youth with emotional and behavioral issues *Measures*: 75 interviews, 33 clinical record reviews, 12 treatment team observations, and reviews of policy and procedure manuals *Limitations*: Insufficient methodological information			Eight Medicaid-funded residential settings in Florida including a statewide inpatient psychiatric program, therapeutic foster care, and therapeutic group care. *External factors:* High rate of dependent children and youth in out of home mental health treatment programs and recognition of need for TIC in Florida	The sites studied were found to have varying levels of TIC in their programming. The most successful demonstrated organizational readiness; competent trauma-informed organizational, clinical, and milieu practices; & youth and family engagement in TIC *Emphasized:* Leadership commitment Model selection Workforce transformation Community inclusion
Martin, Krieg, Esposito, Stubbe, & Cardona (2008) Reduction of restraint and seclusion through collaborative problem solving: A 5-year prospective inpatient study	Collaborative Problem Solving (CPS) Sees child aggressive bx stemming from lagging cognitive skills in the areas: executive functioning, language processing, emotion regulation, cognitive flexibility, and social skills	Prospective study *Sample*: 755 children hospitalized between 2003-2007 *Measures*: Seclusion, restraint, duration, staff injuries *Limitations*: Unable to pinpoint variables responsible for S/R reduction; no empirical measures of aggression; no info on psychotropic meds; no systematic data on child injuries; client injury data limited; no objective measures of adherence to CPS; no assessment of staff, children or family perceptions			Fifteen-bed psychiatric inpatient unit for school age children *External factors:* Federal legislation to reduce restrictive interventions; local investigations into deaths related to restraint and seclusion; condemnation of S/R by all major child serving professional organizations	37.6 fold reduction in restraint and a 3.2 fold reduction in seclusion. Mean duration reduced from mean 27 to mean 21 min per episode Black and Hispanic children were 4x and 50% more likely than White children, respectively, to be restrained or secluded. IQ may have been a confounder Restraint reduction was a more achievable initial target for improvement. Changes maintained despite acuity *Emphasized:* Community inclusion Model selection
Rivard, Bloom, McCorkle, Abramovitz (2005) Implementing a trauma recovery framework for youths in residential treatment	Sanctuary Model: Treatment environment is core modality for modeling healthy relationships among community members. Uses SELF framework (Safety, Emotional mgmt., Loss, Future) Aimed at reducing complex trauma sx among youth in residential settings	Comparison group design, measurement intake, discharge, 6 months *Sample*: Youth sample (*N* = 158) *Measures*: youth demographics, COPES; CBCL, TSC-Children; Rosenberg Self Esteem Scale, Nowicki-Strickland Locus of Control Scale; Inventory of Parent and Peer Attachment; Youth Coping Index; Social Problem Solving Questionnaire *Limitations* use of 3- month youth self-report measures which may not be sensitive to change			Sixteen residential treatment units for adolescents: 4 self-selected; 4 were randomly assigned; 8 units usual services comparison group *External factors:* Large nonprofit mental health and social service agency seeking to better meet trauma needs of children and families it serves—including children with serious emotional disturbance	Sanctuary units outperformed comparison units on COPES scale (see Mechanisms for Improvement, left) Few changes observed in youth outcomes, but Sanctuary unit youths showed ↓ verbal aggression and ↑sense of control over their lives compared to service as usual youths *Emphasized* Workforce transformation Outcome orientation
Russell, Maher, Dorrell, Pitcher, & Henderson (2009) A comparison between Devereux’s safe and positive approaches training curricula in the reduction of injury and restraint	Safe and Positive Approaches (SPA): Comprehensive, multi-component crisis intervention and intervention training program designed to equip staff with knowledge and ability to safely and effectively prevent, de-escalate, and manage crisis situations	*Sample*: Six programs over 6 years; n = 6361 Data analyzed by quarter rather than unique child *Measures*: All restraint rate, rate of prone restraints, youth restraint related injuries; staff restraint related injuries *Limitations*: Variability in definitions regarding restraints and types of restraints and thus data inconsistency			Six residential programs providing treatment to children & youth w/at-risk behaviors, emotional and behavioral disorders, involvement in the criminal justice system, and intellectual and developmental disabilities *External factors* Regulatory policies at center, state, and federal levels	Restraint rates, prone restraint rates, youth injury rates, staff injury rates lower for SPA users than for comparison group *Emphasized* Model selection Workforce transformation

## Analysis of evidence and theory testing

We coded passages of articles on inpatient and residential youth trauma informed care initiatives that related to contexts, mechanisms of action, and outcomes, analyzing patterns in the data related to the program theory articulated in Fig. 2. We also annotated passages of text which disconfirmed our theory or which mentioned important elements of implementation which fell outside these categories. Our ultimate goal was to test and refine our program theory, which insinuates a somewhat stepwise progression from: (1) *including community* in the trauma informed care initiative; (2) supporting *leadership commitment* to TIC; (3) *selecting a TIC model*, intervention, or approach; (4) *transforming the workforce* through hiring the right people, training them, coaching them, and providing them ongoing supervision; (5) promoting an *outcome orientation* by collecting and regularly sharing TIC outcomes and by improving outcomes through plan, do, study, act cycles (PDSA); and finally (6) concretizing TIC structurally and thus ensuring its continued *shared maintenance*.

## Results

The literature examined for this realist review of trauma informed care in inpatient and residential youth settings emphasized the reduction of physical coercion in routine psychiatric and residential care. For example, 9 of 13 reviewed studies [20, 34, 40, 42–47, 49] had as a key aim the reduction or elimination of seclusion and/or restraint, while several studies measured patient and staff injury rates [34, 47, 49]. All nine studies demonstrated targeted reductions in these outcomes, underscoring their potential effectiveness, especially given a set of conditions which would promote successful implementation. Below, we examine elements of implementation thought to have been critical to achieving these outcomes.

### Keys to successful implementation of trauma informed care in youth settings

After extracting and systematically analyzing data, we observed five main factors in our analysis of cross-site TIC implementation: (1) the critical importance of senior leaders prioritizing TIC [21, 40, 42, 44, 46, 47], especially as staff adjust to new ways of working; (2) the necessity of supporting staff by delivering advanced training on the neurobiology and behavioral sequelae of trauma and providing ongoing supervision, coaching, and debriefing of seclusions, restraints, and patient/staff injuries [20, 34, 40–43, 45–47, 49]; (3) the power of listening to patients and families about their experiences, needs, and priorities in the treatment process [21, 42, 47, 48]; (4) the importance of reviewing data and outcome indicators to motivate continued improvement [20, 40, 44–46, 48]; and finally, (5) the need to align policy and practice, formal and informal, with the overarching principles of trauma informed practice [21, 40, 44–46, 48]. After describing these five factors in greater detail below, we discuss our original implementation-science informed TIC program theory model and suggest changes to the theory based on this review.

### Senior leaders prioritizing trauma informed care

Successful TIC implementation requires that organizational leadership, especially senior leaders, be visibly committed to the change process. This means that leaders change their own leadership practices to highlight organizational commitment and support for TIC [33, 47]. Across trauma informed care initiatives, staff knew TIC was a priority by the way leaders behaved. Senior leaders made TIC a standing item in high level meetings, allocated resources, set clear targets, communicated the rationale for the initiative with staff, and articulated “an unwavering belief” that TIC goals were achievable.

In their implementation of the Six Core Strategies, Caldwell et al. [42] underscored the importance of leaders in championing organizational change,

Rigid thinking and old-school mindsets of staff can result in minimal change. Leadership is key to addressing the rigid thinking and mindset of staff and should be outcome-focused to send the message to the organization that culture change is going to happen, the program is changing, and that staff can be part of this change or not (36).

Similarly, executives and leaders at Damar Services, a large residential treatment center, endorsed the agency’s shift to restraint elimination and modeled for staff that the shift in philosophy was not only “part of Damar’s new philosophy, but was the right thing to do as consistent with research and best practice for long-term outcomes” (5) [47]. Finally, two studies underscored the impact leaders can have on the success of TIC by conducting a thorough needs assessment and formulating a clear plan for implementation to guide the organization in achieving goals [21, 46].

### Supporting staff

While implementation science [50] stresses the importance of coaching over one-off training, most TIC frameworks and models in this review urged comprehensive staff training to help staff understand the purpose of TIC and to develop staff buy-in. Specifically, psychoeducation on the neurological and behavioural impacts of trauma was found to be critical [20, 41, 48]. The Risking Connection model and the Sanctuary model deliver curricula via a comprehensive staff-training module. Post-training measures demonstrated changes in staff knowledge, beliefs, and behaviour, although particulars were not reported. Furthermore, studies indicated that training is important because it gives staff common language to use regarding patient experiences and particular trauma informed interventions to be used with patients [33, 41].

Beyond training, studies included in this systematic review indicated the importance of staff members feeling supported throughout the change process. Recertification, ongoing training, coaching, and supervision reinforced trainings and provided staff support. For example, in a large residential facility in Indiana [47], a “resource team” was trained in behavior management and intervention techniques, with recertification required every 3 months. Additional trainings on best practices were provided for all employees of the facility, with direct-care staff required to be recertified in verbal de-escalation techniques every 6 months. In a study of the Attachment, Regulation and Competency (ARC) model [46], which produced a 50% reduction of restraint occurrences within the first 6 months of ARC implementation, researchers discovered that “Staff trauma responses impact staff and clients, as staff may be less able to effectively support and intervene with clients who are experienced as frightening or particularly difficult, as well as difficulty intervening *all* clients, because with of hypervigilance/hyperarousal” (683) [46]. Staff education included training in the Child–Adult Relationship Enhancement (CARE) model adapted from Parent Child Interaction Therapy (PCIT) to ensure staff trainings were “both didactic and experiential.” Trainings included hands-on opportunities for staff to practice self-regulation techniques and focused on the “developmental impact of trauma, building secure attachments, increasing self-regulation and competency, and self-care and vicarious trauma” (684) [46].

### Listening to patients and families

Most models included in this systematic review encourage the inclusion and participation of children and family members in care planning and treatment decisions. Although this element of successful TIC implementation seemed to occupy a less central role in the literature than we hypothesized (or was omitted from author discussion), consultation with patients and families was nonetheless discussed in depth by some authors.

For example, Caldwell et al. [42] reported that including youth and family was central to their success in preventing seclusion and restraint (see Table 3). This was, in part, because researchers and implementers invited youth to share their experiences of restraint with staff. Youth reported that restraint resulted in a loss of self-respect and dignity and in feeling less safe when watching peers. Holstead and colleagues [47] also involved patients in staff training so that staff could hear patients’ experiences of being restrained. In the development of their Trauma Informed Training Curriculum, Hummer et al. [21] emphasized child and youth choice and control, power sharing, collaboration, and caregiver involvement. Lebel et al. [33] suggested involving children and youth in debriefing critical incidents. Finally, the ARC model teaches family members psychoeducational, relational, and regulation techniques so that they can continue to use these skills when the child or youth is eventually discharged from the facility [46].

### Adopting a data and outcomes orientation

Across TIC implementations, an outcome orientation was promoted through regular data sharing in grand rounds and staff meetings. Across implementations, data comprised seclusion and restraint incidents, staff and patient injury rates, and diagnostic and functional symptom prevalence and severity. Data sharing was particularly germane to seclusion/restraint reduction initiatives. For example, Azeem et al. [40] report that outcomes were achieved and maintained by establishing seclusion and restraint reduction targets and goals, collecting and sharing real time data with units so they could monitor progress, encouraging friendly competition between units, and rewarding superior performance—both individually, via performance reviews, and collectively, by unit reviews. In complex initiatives, clinical improvements were also shared with staff to motivate them [51].

### Aligning policy and practice with trauma informed principles

Across studies, consistent multilevel effort was required to align the milieu and organizational culture with the explicit principles of the chosen TIC model or philosophy. One way to bring about change of sufficient magnitude is to adopt a “therapeutic community” approach, such as the one promoted by the Sanctuary Model. In the therapeutic community model, the environment and culture of the organization are therapeutic tools themselves [51]. For example, organizations implementing TIC are encouraged to change the physical environment of the unit to make the treatment space feel safe and welcoming for both patients and staff [33]. Reviewed studies also suggested that trauma-informed principles be included in mission and vision statements, and that such statements be posted visibly to serve as reminders of TIC goals [21, 33].

With regard to changing organizational culture, Goetz & Trujillo [44] found that common challenges to successful implementation of their Patient Focused Intervention Model included troubleshooting staff opposition to longer times required to manage episodes of aggression, for example, through a “show of support” vs. a “show of force.” Eventually, “going hands on” came to be viewed as a de-escalation failure, indicating significant change in the culture of the unit. Additionally, Greene et al. [45] summed up the process required to align their model, Collaborative Problem Solving, with unit policies and practices:

The staff examined many long-standing unit policies and procedures, such as expectations for patient participation in therapy groups, visitation hours and policies, the grouping of patients, and staffing patterns, and worked together to improve compatibility between the unit structure, the primary goals of stabilization and assessment, the staff, and patients (612).

Findings from this review suggest that allocating process time for the slow and organic changes that must take place to accommodate the new way of practicing should be factored into TIC implementation plans.

### Limitations

Limitations of this systematic review included: (1) a truncated five-step realist review process in which we were unable to contact authors of all studies chosen for inclusion in the review to gather additional information about implementation context, mechanisms, and outcomes; (2) little description of our efforts to engage in knowledge translation with key stakeholders involved in the project of which this review was an initial component; and (3) no quantitative threshold for program/intervention quality/assessment of bias. Findings of the review should thus be approached with scepticism and applied with caution.

## Discussion

The purpose of this systematic review was to answer the question, “What is it about trauma-informed care that works, for whom, in what circumstances, in what respects, and why?” We hypothesized that successful implementation—defined as the achievement of specified TIC targets such as reduced episodes of seclusion and restraint, fewer staff and patient injuries, and greater patient and staff satisfaction, for example—would closely mirror the implementation science-based steps articulated in the best known S/R reduction intervention, the Six Core Strategies to Reduce Seclusion and Restraint. These strategies are: (1) leadership towards organizational change; (2) using data to inform practice; (3) workforce development; (4) use of seclusion/restraint prevention tools; (5) consumer roles in inpatient settings; and (6) debriefing techniques. Cross-matched with the NIRN implementation drivers in our program theory, these became: (1) community inclusion; 2) leadership commitment; (3) model selection; (4) workforce transformation; (5) outcome orientation; and (6) shared maintenance.

### Program theory revision

**Fig. 3:**
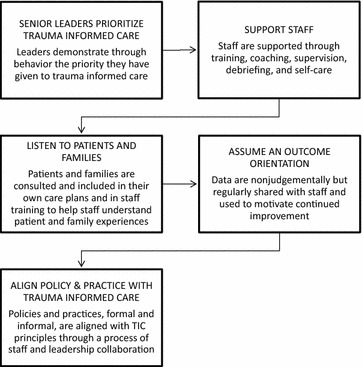
Revised program theory of trauma informed practice implementation

We found two large discrepancies between our original program theory model and the data we analyzed systematically: (1) the sequence of implementation activities undertaken, particularly activities to ensure patient and family participation and 2) the importance of choosing a particular program model (see Figs. 2, 3).

First, in our original program theory, community inclusion was viewed as a precursor to leadership commitment. That is, we speculated that successful trauma informed care would require that leaders of residential treatment centers, mental health agencies, health systems, and hospitals would first consult patients and families before deciding what changes to make or what model to adopt. However, overall, only a few authors [21, 42, 47, 48] discussed consultations with patients and families. Notably, when community inclusion was discussed, it had a very positive impact on the initiative—especially when patients spoke directly to staff about their lived experience.

Second, in our original program theory, we asserted that choosing a comprehensive evidence informed practice model would aid successful TIC implementation, especially when implemented with fidelity. But in fact, although some authors emphasized the salience of their particular model and credited it with changes which were achieved in staff and milieu behavior [20, 21, 41, 48] almost half the studies we reviewed reported efforts solely intended to reduce seclusion and restraint [34, 40, 42–44, 49]. What’s more, despite a critique of standalone S/R reduction initiatives, these approaches produced significant reductions in episodes of seclusion and restraint, as well as staff and patient injuries (See Table 3). Following are brief profiles of two initiatives that represent these poles of trauma informed care: comprehensive TIC models and primary S/R reduction models.

In their discussion of Collaborative Problem Solving (CPS), a comprehensive model, Greene et al. [45] assert that reducing restrictive measures is not enough. Instead, a theoretically-based model should be adopted, as theory builds analytic capacity and increases staff understanding of the difficult behaviors they will encounter when working with children and youth:

Although reducing the use of timeout, quiet room time, restraint, and seclusion is an important goal, focusing on that specific goal alone is unlikely to accomplish the mission. Rather, we have found that even with a strong commitment from unit leadership to reduce or eliminate such practices, staff must also be provided with a comprehensive model of care, including a common set of assumptions about the factors underlying children’s aggressive or unsafe behavior, an understanding that the manner in which limits are set and expectations pursued by adults may precipitate such behavior, and an emphasis on crisis prevention rather than crisis management. In this view, reduction in the use of physically restrictive procedures is an outgrowth of good care, not necessarily an endpoint in and of itself (611).

Outcomes for this CPS implementation were very positive: Seclusion and restraint episodes declined from 281 in the 9 months before training to one incident 15 months post-training. Additionally, staff and patient injuries declined from an average of 10.8 per month to 3.3 per month.

On the other end of the spectrum, a modest UK intervention, Post Restraint Reduction Meetings, was quite straightforward [43]. Reduction Restraint workshops were delivered to all staff for children’s homes and residential settings. Additionally, reduction restraint meetings were held within 72 h of each restraint, and coaching from researchers was made available to staff. This intervention, exclusively focused on restraint reduction, achieved a 32% decrease in restrictive physical interventions—with the greatest reduction observed in the most restrictive supine floor restraints. These findings are consistent with those of Martin et al. [34], who suggest that “restraint may be a more achievable first target of reduction efforts” than seclusion or other targets for improvement (1409).

### Implementation challenges and lessons learned

In the course of systematically reviewing studies, a pattern emerged in which the larger scale organizational cultural changes attempted by comprehensive models—which require more resources on the front end—perhaps produced longer-term and ‘deeper’ changes to organizational culture. This finding is consistent with implementation science literature, as ‘deeper’ organization change requires repeated and direct confrontation with “adaptive challenges” versus “technical problems” [51].

As discussed below, TIC initiatives may benefit from allocating dedicated staff time so that those implementing TIC are not recruited into “old” ways of doing practice or torn between roles because of time and resource allocation or role conflict. For example, discussing implementation of the Fairy Tale model in a particular agency, Greenwald et al. [20] noted,

It is the first author of this study’s impression that this agency’s therapists adopted the Fairy Tale model more slowly and incompletely than any other training cohort in recent years. This…seemed to be a direct consequence of having competing roles. When we have trained other therapists with similar dual roles, they had similar difficulty. The therapists’ time and role definition must be protected so that they are able to provide the treatment (150).

Articles reviewed suggested that to achieve a trauma informed organizational milieu which embodies patient choice, collaboration and control, organizations may confront long-standing issues like power struggles, the culture of psychiatry, and perceived efficiencies of using physical and chemical seclusion and restraint versus interventions that require substantial time and skill (e.g., collaborative problem solving). Brown et al. [41] found that staff who were trained in the Risking Connection model and *who trained other staff* showed sustained positive changes in knowledge, beliefs, and behaviors. Although not definitive, a train-the-trainer rather than purveyor model may produce financial efficiencies and generate longer term change in organizational culture.

## Conclusion

Taken together, data from this review suggest that trauma informed care initiatives which are comprehensive, theoretically grounded, and developmentally-informed and which seek to align all facets of treatment with the principles of safety, choice, and collaboration may reduce seclusion, restraint, and staff and patient injury rates. They may also add value by improving clinical outcomes. Similarly, quality assurance efforts to reduce costly, poorly evidenced, and potentially injurious and coercive physical interventions may result in significant positive changes in youth serving environments.

Given the broad array of age, developmental needs, and clinical presentations in child and youth inpatient and residential settings, as well as the investment required to effect overall systems change, trauma informed care may best be implemented on a unit-by-unit or agency-by-agency basis. That is, some units or agencies may opt to target coercive events while others may choose to implement theoretically-based models whose primary benefits include change in symptom expression and severity and whose secondary benefits include reductions in injuries and traumatic stress. Both approaches show promise. However, the state of science regarding trauma informed care is quite underdeveloped. To advance the field, additional research should seek to demonstrate, longitudinally, the efficacy of both types of efforts in improving patient safety and long term treatment outcomes.

## Additional file

**Additional file 1: Table S1.** Mechanisms of action in successful TIC implementations.

## Abbreviations

TIC: trauma informed care
TIP: trauma informed practice
ACE: adverse childhood experience
NCTSN: National Child Traumatic Stress Network
ARC: attachment, self-regulation, and competency
6CS: six core strategies
CPS: collaborative problem solving
RC: risking connection
PDSA: plan, do, study, act
CARE: Child-Adult Relationship Enhancement
PCIT: Parent Child Interaction Therapy (PCIT)
NASHMHPD: National Association of State Mental Health Program Directors
