# Supplementary material for: What are effective strategies for implementing trauma-informed care in youth inpatient psychiatric and residential treatment settings? A realist systematic review
Source: Int J Ment Health Syst. 2017 May 11;11:36. doi: 10.1186/s13033-017-0137-3 (PMC5425975; doi:10.1186/s13033-017-0137-3)
Supplement: Supplementary file 1 — Additional file 1: Table S1. Mechanisms of action in successful TIC implementations. [file 13033_2017_137_MOESM1_ESM.docx]

Additional File 1

TableS1: Mechanisms of Action in Successful TIC Implementations

| STUDY TITLE | MECHANISMS OF ACTION |
| --- | --- |
| Azeem, Aujla, Rammerth, Binsfield, & Jones (2011)  Effectiveness of Six Core Strategies Based on Trauma Informed Care in Reducing Seclusions and Restraints at a Child and Adolescent Psychiatric Hospitaljcap**_262 11..15** | - Senior mgmt. made goal to reduce seclusion/restraint - Shared reasons w/ staff - Allocated resources - Standing agenda item-S/R - Goals/targets established; units encouraged to compete - Grand rounds & staff mtgs used to meet targets - Best practices rewarded - Additional training as needed - Nonjudgmental review of S/R - All new staff oriented on neuro/bio/psycho effects of trauma - Job descriptions, competencies, performance evals include TIP - Preventive tools to reduce S/R - Consumer feedback invited |
| Brown, Baker, & Wilcox (2012)  Risking Connection Trauma Training: A Pathway Toward Trauma-informed Care in Child Congregate Settings | - Unlike other TIC models, this intervention sought to implement a theory-based training (RC) to effect system-wide adoption of TIC. - RC training emphasizes ‘the self of the treater,’ focusing on vicarious traumatization and countertransference. - Emphasizes care of both client and treatment provider. - RC uses a train-the-trainer (TTT) model of dissemination, hypothesized to increase organizational capacity and staff buy-in. |
| Caldwell, et al., 2014  Successful Seclusion and Restraint Prevention Efforts in Child & Adolescent Programs | - Leaders ‘strongly emphasized primary prevention’ - S/R standing agenda in all weekly staff meetings (all sites) - Leaders articulated ‘unwavering belief’ that goals of eliminating S/R were achievable - Leaders learned that core strategy of leadership ‘depends on what executive leaders truly believe’ and ‘the willingness to make a whole-hearted commitment to those beliefs’—especially to overcome ‘old school’ staff mindsets - Dashboards of S/R for each unit/facility shared in real time - Including youth and family key to success in preventing S/R; family visit any time - Site #1-trainings emphasized person-centered care; in Site #2-‘hands off’ facility rule passed and only hugs given-no restraints; Site #3 eliminated point system, reducing S/R - ASAP team provided support to staff who experienced trauma - Individualized tx plan - Sensory tools used (pet therapy, visits to animal shelter, music therapy, cooking, swimming) - Debriefing (staff and youth) focuses on chain analysis - Youth reported that restraint resulted in a loss of self-respect and dignity and in feeling less safe when watching peers |
| Deveau & Leich, 2014  The impact of restraint reduction meetings on the use of restrictive physical interventions in English residential service for children and young people | - Reduction Restraint workshop delivered to all staff - Reduction Restraint meetings to be held within 72 hrs of each restraint - Information distributed to young people, staff, and professionals - Additional coaching/contact with researchers if requested |
| Goetz & Trujillo, 2012  A change in culture: Violence prevention in an acute behavioral health setting | - Two day workshop with Beth Caldwell, director of SAMHSA/Center for MH Svcs - Initial psychosocials included questions about past trauma, triggers that evoke anxiety, coping skills inventory - Power struggles reduced - Train the trainer approach with Sorenson & Wilder Associates aggression mgmt. program - Major change in time taken to manage episodes of aggression (rather than moving to subdue aggression)—“show of support” instead of “show of force” - “Going hands-on” seen as de-escalation failure - Nurse Quality Specialist, mgmt. and safety committee analyzed monthly data on S/R - Daily leadership reviews of S/R initiated to involve more staff |
| Greene, Ablon, & Martin (2006)  Use of collaborative problem solving to reduce seclusion and restraint in child and adolescent inpatient units | - Even with a strong commitment from unit leadership, staff must be equipped with a model of care - CPS teaches adults to solve problems and has implications for staff-staff relationships and milieu overall - Many positive changes on unit were “a byproduct of encouraging unit staff and leadership to discuss mechanisms for altering the culture of the unit” - Examination of long-standing unit policies and procedures, such as expectations for patient participation in therapy groups, visitation hours, staffing patterns - Milieu and supervisors began reviewing difficult interactions with patients to identify triggers of aggressive behavior |
| Greenwald, Siradas, Schmitt, Reslan, Fierle, & Sande (2012)  Implementing trauma-informed treatment for youth in a residential facility: First-year outcomes | Phases of Treatment include:   - Evaluation including learning about child’s strengths, trauma/loss history, presenting problems; - Identification & enhancement of client’s goals & motivation; - Trauma-informed case formulation and treatment contracting; - Stabilization, including case mgmt., parent/staff training, problem solving, avoidance of high risk situations; - Identification & enhancement of coping and affect tolerance skills; - Resolution of trauma and loss memories; - Consolidation of gains; and - Anticipation of future challenges.   Training includes motivational interviewing and EMDR components |
| Hodgdon, Kinniburgh, Gabowitz, Blaustein, & Spinazzola (2013)  Development and implementation of trauma-informed programming in youth residential treatment centers using the ARC framework | Exploration & Adoption Stage   - Identify key stakeholders - Conduct trauma-informed needs assessment   - Physical environment should reflect TIP   - Trainings on developmental impact of trauma   - Staff support & self-care practices   - Integration of services, regular & routine communication   - Milieu culture changed to view problem behaviors as attempts to manage trauma symptoms   Program Installation   - Build implementation team - Train program staff on impact of trauma, assessment, intervention   Initial Implementation   - Implement milieu bx enhancement initiatives - Implement EBP Individual & group therapy   Sustainability   - Sustain trauma-informed svcs |
| Holstead, Lamond, Dalton, Horne, & Crick (2010)  Restraint reduction in children’s residential treatment facilities: Implementation at Damar Services | - Formed a Resource Management Team that was trained in behavior management and intervention - Recertified every months - Team member coached staff to de-escalate situations - Employee training: Each experienced a restraint as part of training, and staff heard from patients who had experienced restraint. - In 2008, agency declared itself restraint free |
| Hummer, Dollard, Robst, & Armstrong (2010) | - Systematic debriefings of S/R - Policies and procedures ensuring children and youth knew program expectations - Child & youth choice and control - Collaboration, power sharing, empowerment - Caregiver involvement - Preparation for placement transition - Formal service policies - Trauma screening, assessment, service planning - Administrative support for program-wide trauma-informed services - Staff training & education - Human resources practices |
| Martin, Krieg, Esposito, Stubbe, & Cardona (2008)  Reduction of restraint and seclusion through collaborative problem solving: A 5-year prospective inpatient study | - Identify pertinent social and cognitive pathway impairments and precipitating antecedent events to child aggression - Models alternative means of de-escalation through social problem solving, conflict resolution, anger mgmt. - 3 hour staff training followed by 90-minute supervision with developers of CPS |
| Rivard, Bloom, McCorkle, Abramovitz (2005)  Implementing a trauma recovery framework for youths in residential treatment | - Community members help and support each other - Program encourages open expression of feelings - Program promotes self-sufficiency and independence in decision making - Community members seek to understand their feelings and personal problems - Program environment promotes physical, social, and psychological safety for staff and clients |
| Russell, Maher, Dorrell, Pitcher, & Henderson (2009)  A comparison between Devereux’s safe and positive approaches training curricula in the reduction of injury and restraint | - Accountable crisis management that is based on prevention and positive supports and an effective curricula   - Staff effectiveness training   - Safety techniques training   - Personal emergency interventions training   - Supervision of safe and positive approaches |
